# Supplementary material for: Independent regulation of age associated fat accumulation and longevity
Source: Nat Commun. 2020 Jun 3;11:2790. doi: 10.1038/s41467-020-16358-7 (PMC7270101; doi:10.1038/s41467-020-16358-7)
Supplement: Supplementary file 1 — Supplementary Information [file 41467_2020_16358_MOESM1_ESM.pdf]

Independent regulation of age associated fat accumulation and longevity

Beas et. al.

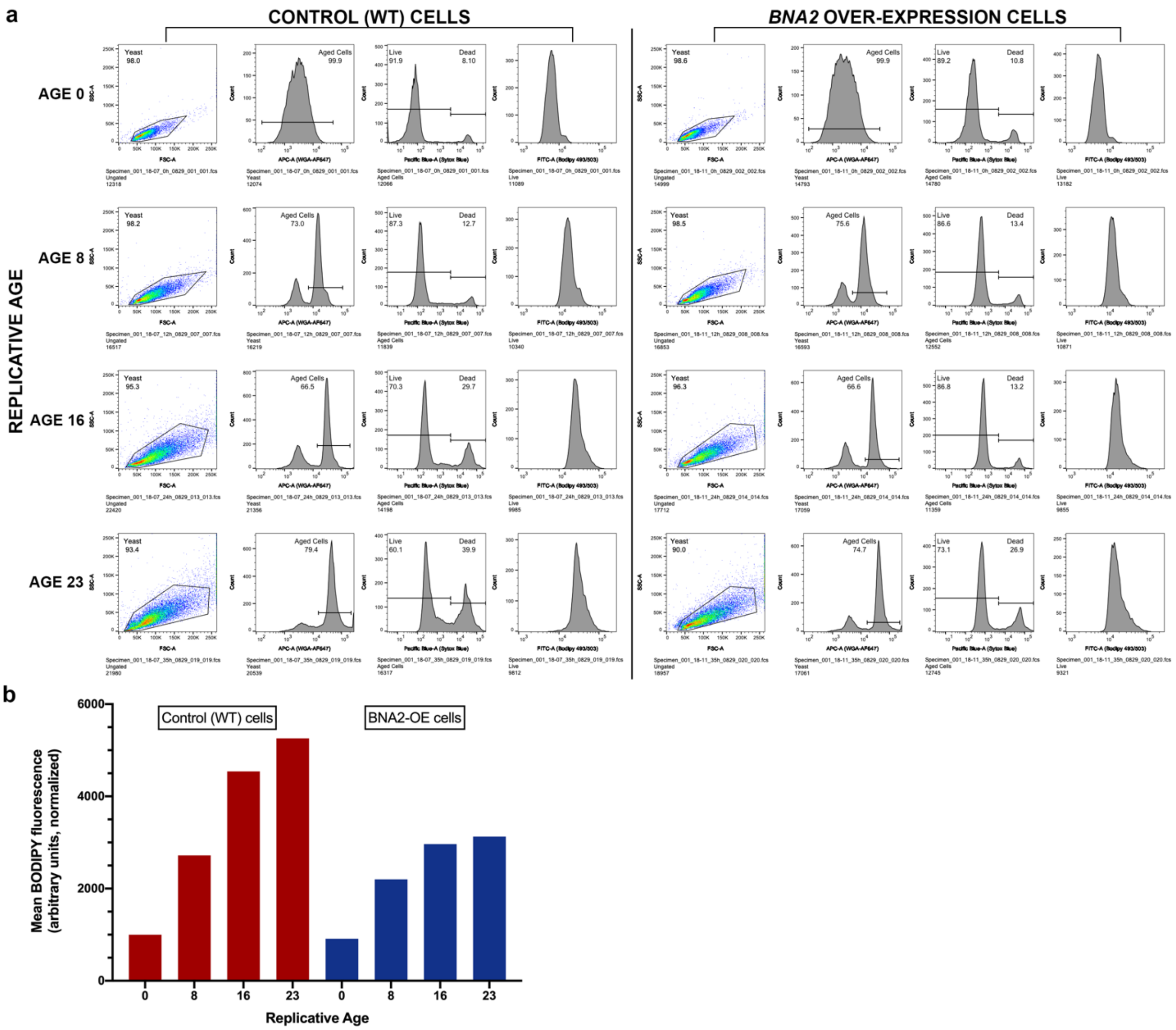

Supplementary Figure 1: Quantification of lipid droplet levels by flow cytometry. a, An example analysis of 1 of 13 biological replicates included in Figure 1d showing how lipid droplet (LD) levels were quantified using BODIPY (493/503) staining in young and aging cells (from top to bottom panels). Aged Control (WT, AB18-07, left) and *BNA2* over-expression (*BNA2*-OE, right) mother cells enriched from cultures (see Methods) were stained, and fluorescence was measured in multiple channels and fluorescent filters by flow cytometry using the gating strategy shown in **a**. From left to right: 1) forward and side scatter of cells for size and complexity (FSC-A versus SCC-A), 2) wheat germ agglutinin (WGA, binds bud scars) Alexa Fluor 647 (WGA-647) staining distinguishes aged mother cells (WGF-647 binds budscars, and signals detected) versus daughter cells (daughter cells have no budscars, background signals detected) (APC-A channel), 3) Sytox Blue stain permeates dead cells, distinguishes stain-positive dead cells and stain-negative live cells (PacificBlue-A channel), and 4) BODIPY signals quantification in live, aged mother cells (FITC-A). Measurements analyzed using FlowJo v.10.6.1 software (Becton, Dickinson & Company, Franklin Lakes, NJ). **b,** Example plot showing mean (normalized) BODIPY signals quantified in "a". To normalize, all BODIPY measurements quantified from live, aged cells for every age and strain were 1) divided by the mean BODIPY signal calculated for live, age 0 Control (WT) cells, and 2) multiplied by 1000. The normalized signals for each strain and time point were then averaged and plotted (Control (WT), red bars; *BNA2*-OE cells, blue bars) using

GraphPad Prism 8 v 8.3.1 (San Diego, CA). Source data for a-b are provided in the Source Data file. For all flow cytometry based figure panels (Figures 1d, 2b-g, Supplementary Figure 2a), the same strategy was used to determine the mean, normalized BODIPY signals per time point for each strain within each individual experiment, and these signals were then used to calculate the overall average BODIPY signal for each age and strain over all biological replicates and to generate scatter plots shown in figure panels. Source data for a-b are provided in the Source Data file.

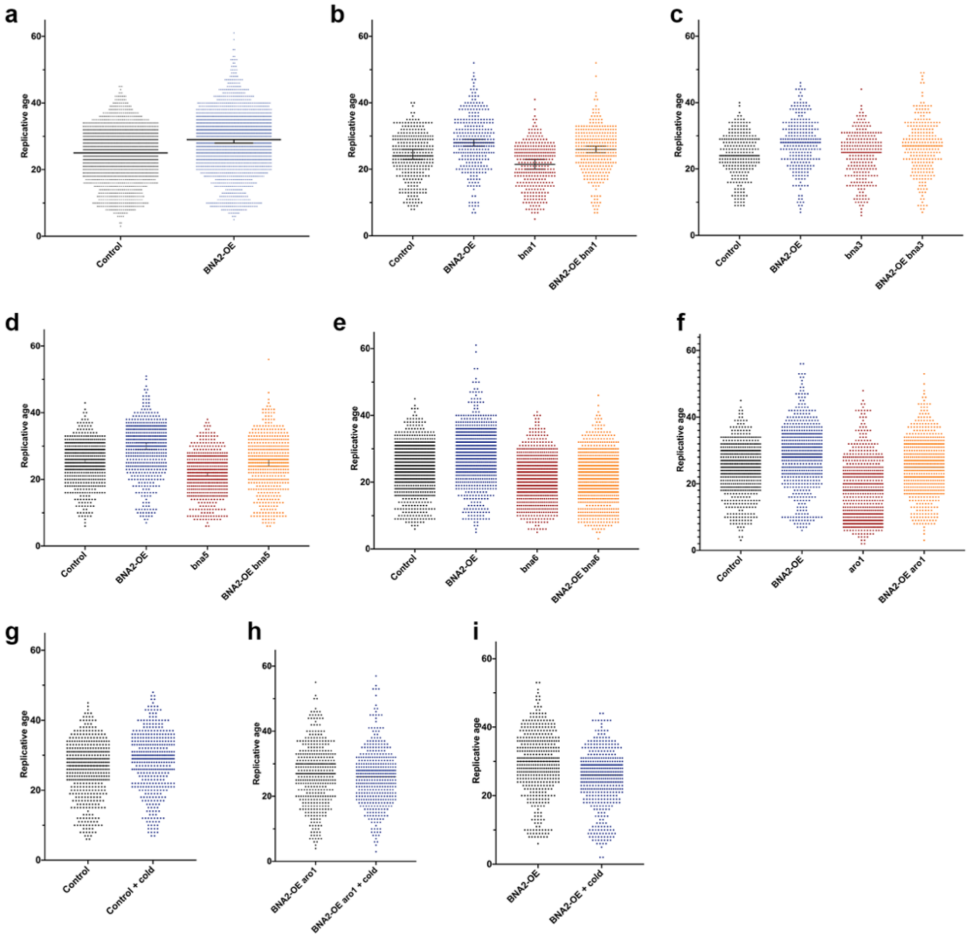

Supplementary Figure 2: Replicative ages of control and mutant strains. Data from Kaplan-Meier plots in the main text figures were replotted as distributions. Data: number of cells; median age; mean age ± SEM. **a,** Control cells (black stars): 2125; 25; 24.55 ± 0.16. *BNA2*-OE cells (blue stars): 2275; 29; 28.36 ± 0.19. **b,** Control cells: 300; 24; 23.8 ± 0.4. *BNA2*-OE cells: 300; 28; 28.31 ± 0.48. *bna1Δ* cells (red stars): 300; 21.5; 20.91 ± 0.39. *BNA2*-OE *bna1Δ* cells (orange stars): 300; 26; 25.61 ± 0.41. **c,** Control cells: 250; 24; 23.98 ± 0.42. *BNA2*-OE cells: 250; 28; 27.64 ± 0.49. *bna3Δ* cells (red stars): 250; 24; 23.03 ± 0.45. *BNA2*-OE *bna3Δ* cells (orange stars): 250; 27; 26.66 ± 0.51. **d,** Control cells: 475; 25; 24.89 ± 0.30. BNA2-OE cells: 475; 30; 28.84 ± 0.38. *bna5Δ* cells (red stars): 475; 21; 21.24 ± 0.30. *BNA2*-OE *bna5Δ* cells (orange stars): 475; 25; 24.49 ± 0.38. **e,** Control cells: 775; 25; 24.32 ± 0.26. *BNA2*-OE cells: 750; 29; 28.03 ± 0.31. *bna6Δ* cells (red stars): 700; 21; 21.22 ± 0.26. *BNA2*-OE *bna6Δ* cells (orange stars): 725; 22; 21.51 ± 0.28. **f,** Control cells: 550; 25; 24.11 ± 0.33. *BNA2*-OE cells: 550; 29; 28.36 ± 0.41. *aro1Δ* cells (red stars): 600; 18; 18.24 ± 0.38. *BNA2*-OE *aro1Δ* cells (orange stars): 575; 25; 25.29 ± 0.36. **g,** Control cells 30° C (black stars): 425; 27; 25.76 ± 0.40. Control 4° C (blue stars): 400; 29; 27.92 ± 0.43. **h,** *BNA2*-OE *aro1Δ* cells 30° C (black stars): 400; 27; 26.65 ± 0.47. *BNA2*-OE *aro1Δ* + cold cells 4° C (blue stars): 400; 26; 25.97 ± 0.44. **I,** *BNA2*-OE cells 30° C (black stars): 425; 30; 29.24 ± 0.46. *BNA2*-OE 4° C (blue stars): 425; 26; 24.64 ± 0.41. Source data for a-I are provided in Source Data file.

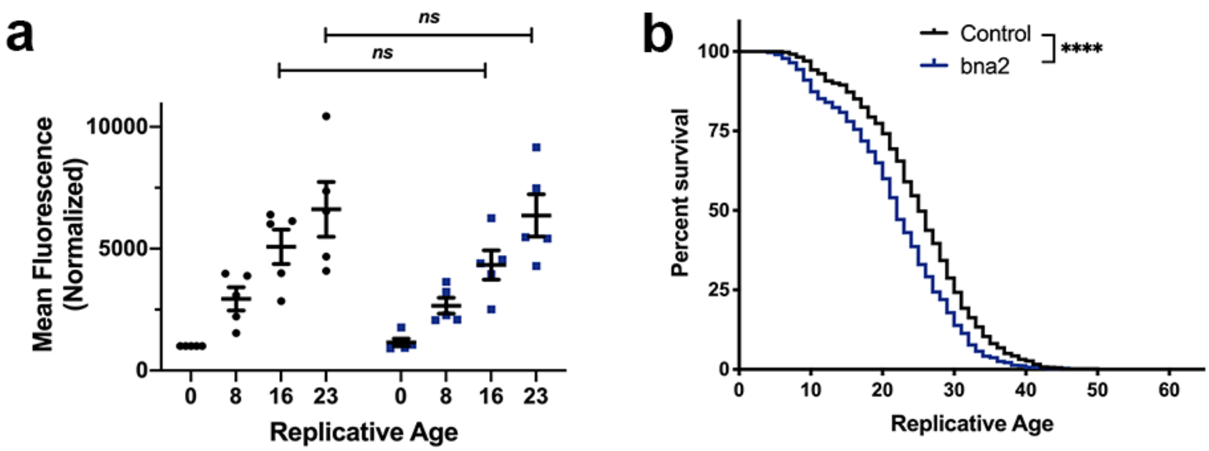

Supplementary Figure 3: *BNA2* deletion decreases lifespan but does not impact lipid droplet accumulation during aging. a, BODIPY signals quantified in Control (WT, black dots) and *bna2Δ* (blue squares) cells were normalized, averaged, and plotted (± SEM) as in Supplementary Figure 1 (n=5 independent experiments). 2-way ANOVA Multiple comparisons: p > 0.05 indicated as ns = not significant (). **b,** RLS analysis of 800 Control cells (black line) and 600 *bna2Δ* cells (blue line) as in Figure 1f over n=6 independent experiments (see Methods). Median and maximal lifespans (± SEM): Control (25.7 ± 0.4, 44.7 ± 1.3), bna2Δ (22.2 ± 0.5, 40.2 ± 2.3). Log rank test: p > 0.05 indicated as ns = not significant; p<0.0001 indicated as ****. Source data for a-b are provided as a Source Data file.

Supplementary Figure 4: Global metabolomics heat map. Source data are provided in Source Data file.

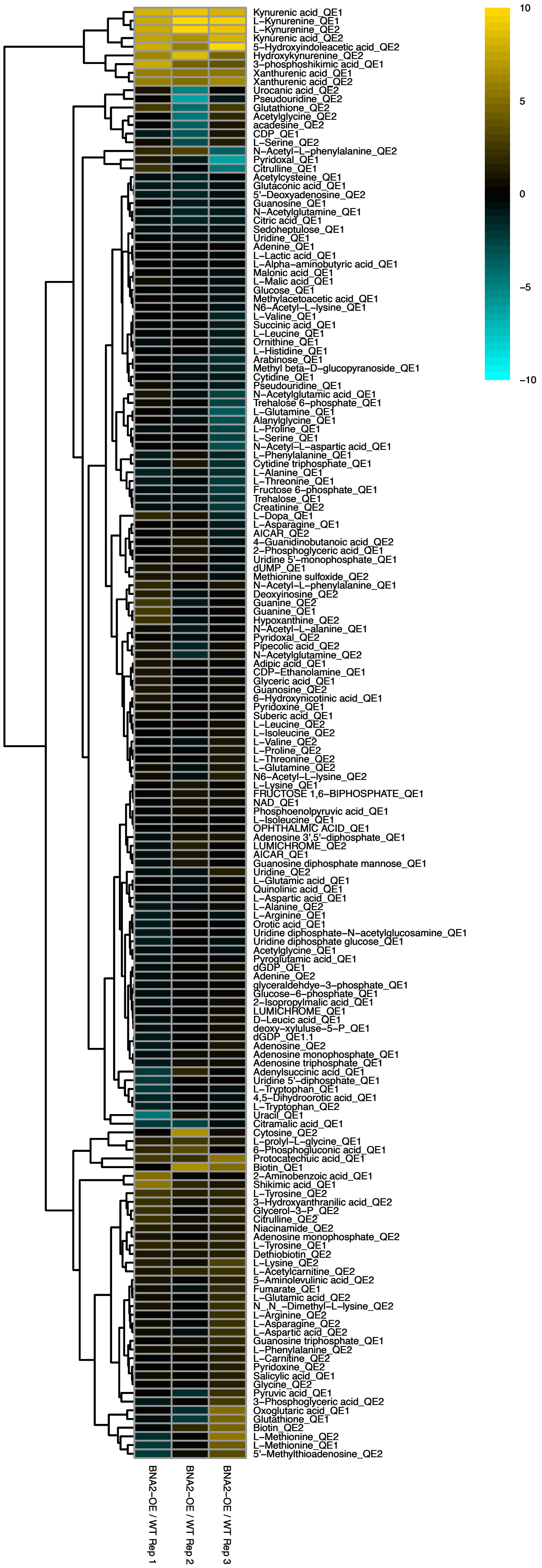

Supplementary Table 1 (strains)

Strain Name	Common Name	Genotype
AB18-07	Control (WT)	MATa/α his3Δ1/his3Δ1 leu2Δ0/leu2Δ0 ura3Δ0/ura3Δ0 lys2Δ0/+ hoΔ::SCW11pr-Cre-EBD78-NATMX/hoΔ::SCW11pr-Cre-EBD78-NATMX loxP-CDC20-Intron-loxP-HPHMX/loxP-CDC20-Intron-loxP-HPHMX loxP-UBC9-loxP-LEU2/loxP-UBC9-loxP-LEU2 GPD-URA3-xsome1/GPD-URA3-chr1
AB18-11	BNA2-OE	MATa/α his3Δ1/his3Δ1 leu2Δ0/leu2Δ0 ura3Δ0/ura3Δ0 lys2Δ0/+ hoΔ::SCW11pr-Cre-EBD78-NATMX/hoΔ::SCW11pr-Cre-EBD78-NATMX loxP-CDC20-Intron-loxP-HPHMX/loxP-CDC20-Intron-loxP-HPHMX loxP-UBC9-loxP-LEU2/loxP-UBC9-loxP-LEU2 GPD-BNA2-URA3-chr1/GPD-BNA2-URA3-chr1
AB19-75	bna5Δ	MATa/α his3Δ1/his3Δ1 leu2Δ0/leu2Δ0 ura3Δ0/ura3Δ0 lys2Δ0/+ hoΔ::SCW11pr-Cre-EBD78-NATMX/hoΔ::SCW11pr-Cre-EBD78-NATMX loxP-CDC20-Intron-loxP-HPHMX/loxP-CDC20-Intron-loxP-HPHMX loxP-UBC9-loxP-LEU2/loxP-UBC9-loxP-LEU2 bna5::KanMx/bna5::KanMx GPD-URA3-chr1/GPD-URA3-chr1
AB19-79	BNA2-OE bna5Δ	MATa/α his3Δ1/his3Δ1 leu2Δ0/leu2Δ0 ura3Δ0/ura3Δ0 lys2Δ0/+ hoΔ::SCW11pr-Cre-EBD78-NATMX/hoΔ::SCW11pr-Cre-EBD78-NATMX loxP-CDC20-Intron-loxP-HPHMX/loxP-CDC20-Intron-loxP-HPHMX loxP-UBC9-loxP-LEU2/loxP-UBC9-loxP-LEU2 bna5::KanMx/bna5::KanMx GPD-BNA2-URA3-chr1/GPD-BNA2-URA3-chr1
AB20-16	bna2Δ	MATa/α his3Δ1/his3Δ1 leu2Δ0/leu2Δ0 ura3Δ0/ura3Δ0 lys2Δ0/+ hoΔ::SCW11pr-Cre-EBD78-NATMX/hoΔ::SCW11pr-Cre-EBD78-NATMX loxP-CDC20-Intron-loxP-HPHMX/loxP-CDC20-Intron-loxP-HPHMX loxP-UBC9-loxP-LEU2/loxP-UBC9-loxP-LEU2 bna2::KanMx/bna2::KanMx GPD-URA3-chr1/GPD-URA3-chr1
AB21-02	bna1Δ	MATa/α his3Δ1/his3Δ1 leu2Δ0/leu2Δ0 ura3Δ0/ura3Δ0 lys2Δ0/+ hoΔ::SCW11pr-Cre-EBD78-NATMX/hoΔ::SCW11pr-Cre-EBD78-NATMX loxP-CDC20-Intron-loxP-HPHMX/loxP-CDC20-Intron-loxP-HPHMX loxP-UBC9-loxP-LEU2/loxP-UBC9-loxP-LEU2 bna1::KanMx/bna1::KanMx GPD-URA3-chr1/GPD-URA3-chr1
AB21-06	BNA2-OE bna1Δ	MATa/α his3Δ1/his3Δ1 leu2Δ0/leu2Δ0 ura3Δ0/ura3Δ0 lys2Δ0/+ hoΔ::SCW11pr-Cre-EBD78-NATMX/hoΔ::SCW11pr-Cre-EBD78-NATMX loxP-CDC20-Intron-loxP-HPHMX/loxP-CDC20-Intron-loxP-HPHMX loxP-UBC9-loxP-LEU2/loxP-UBC9-loxP-LEU2 bna1::KanMx/bna1::KanMx GPD-BNA2-URA3-chr1/GPD-BNA2-URA3-chr1
AB21-11	bna6Δ	MATa/α his3Δ1/his3Δ1 leu2Δ0/leu2Δ0 ura3Δ0/ura3Δ0 lys2Δ0/+ hoΔ::SCW11pr-Cre-EBD78-NATMX/hoΔ::SCW11pr-Cre-EBD78-NATMX loxP-CDC20-Intron-loxP-HPHMX/loxP-CDC20-Intron-loxP-HPHMX loxP-UBC9-loxP-LEU2/loxP-UBC9-loxP-LEU2 bna6::KanMx/bna6::KanMx GPD-URA3-chr1/GPD-URA3-chr1
AB21-14	BNA2-OE bna6Δ	MATa/α his3Δ1/his3Δ1 leu2Δ0/leu2Δ0 ura3Δ0/ura3Δ0 lys2Δ0/+ hoΔ::SCW11pr-Cre-EBD78-NATMX/hoΔ::SCW11pr-Cre-EBD78-NATMX loxP-CDC20-Intron-loxP-HPHMX/loxP-CDC20-Intron-loxP-HPHMX loxP-UBC9-loxP-LEU2/loxP-UBC9-loxP-LEU2 bna6::KanMx/bna6::KanMx GPD-BNA2-URA3-chr1/GPD-BNA2-URA3-chr1
AB21-75	Control Erg6p-mCh	MATa/α his3Δ1/his3Δ1 leu2Δ0/leu2Δ0 ura3Δ0/ura3Δ0 lys2Δ0/+ hoΔ::SCW11pr-Cre-EBD78-NATMX/hoΔ::SCW11pr-Cre-EBD78-NATMX loxP-CDC20-Intron-loxP-HPHMX/loxP-CDC20-Intron-loxP-HPHMX loxP-UBC9-loxP-LEU2/loxP-UBC9-loxP-LEU2 ERG6-yemCh-KANMX/+ GPD-URA3-chr1/GPD-URA3-chr1
AB21-77	BNA2-OE Erg6p-mCh	MATa/α his3Δ1/his3Δ1 leu2Δ0/leu2Δ0 ura3Δ0/ura3Δ0 lys2Δ0/+ hoΔ::SCW11pr-Cre-EBD78-NATMX/hoΔ::SCW11pr-Cre-EBD78-NATMX loxP-CDC20-Intron-loxP-HPHMX/loxP-CDC20-Intron-loxP-HPHMX loxP-UBC9-loxP-LEU2/loxP-UBC9-loxP-LEU2 ERG6-yemCh-KANMX/+ GPD-BNA2-URA3-chr1/GPD-BNA2-URA3-chr1
AB25-68	aro1Δ	MATa/α his3Δ1/his3Δ1 leu2Δ0/leu2Δ0 ura3Δ0/ura3Δ0 lys2Δ0/+ hoΔ::SCW11pr-Cre-EBD78-NATMX/hoΔ::SCW11pr-Cre-EBD78-NATMX loxP-CDC20-Intron-loxP-HPHMX/loxP-CDC20-Intron-loxP-HPHMX loxP-UBC9-loxP-LEU2/loxP-UBC9-loxP-LEU2 aro1::KanMx/aro1::KanMx GPD-URA3-chr1/GPD-URA3-chr1
AB26-23	BNA2-OE aro1Δ	MATa/α his3Δ1/his3Δ1 leu2Δ0/leu2Δ0 ura3Δ0/ura3Δ0 lys2Δ0/+ hoΔ::SCW11pr-Cre-EBD78-NATMX/hoΔ::SCW11pr-Cre-EBD78-NATMX loxP-CDC20-Intron-loxP-HPHMX/loxP-CDC20-Intron-loxP-HPHMX loxP-UBC9-loxP-LEU2/loxP-UBC9-loxP-LEU2 aro1::KanMx/aro1::KanMx GPD-BNA2-URA3-chr1/GPD-BNA2-URA3-chr1
AB26-53	bna3Δ	MATa/α his3Δ1/his3Δ1 leu2Δ0/leu2Δ0 ura3Δ0/ura3Δ0 lys2Δ0/+ hoΔ::SCW11pr-Cre-EBD78-NATMX/hoΔ::SCW11pr-Cre-EBD78-NATMX loxP-CDC20-Intron-loxP-HPHMX/loxP-CDC20-Intron-loxP-HPHMX loxP-UBC9-loxP-LEU2/loxP-UBC9-loxP-LEU2 bna3::KanMx/bna3::KanMx GPD-URA3-chr1/GPD-URA3-chr1
AB26-57	BNA2-OE bna3Δ	MATa/@ his3Δ1/his3Δ1 leu2Δ0/leu2Δ0 ura3Δ0/ura3Δ0 lys2Δ0/+ hoΔ::SCW11pr-Cre-EBD78-NATMX/hoΔ::SCW11pr-Cre-EBD78-NATMX loxP-CDC20-Intron-loxP-HPHMX/loxP-CDC20-Intron-loxP-HPHMX loxP-UBC9-loxP-LEU2/loxP-UBC9-loxP-LEU2 bna3::KanMx/bna3::KanMx GPD-BNA2-URA3-chr1/GPD-BNA2-URA3-chr1

AB27-15	bna7Δ	MATa/α his3Δ1/his3Δ1 leu2Δ0/leu2Δ0 ura3Δ0/ura3Δ0 lys2Δ0/+ hoΔ::SCW11pr-Cre-EBD78-NATMX/hoΔ::SCW11pr-Cre-EBD78-NATMX loxP-CDC20-Intron-loxP-HPHMX/loxP-CDC20-Intron-loxP-HPHMX loxP-UBC9-loxP-LEU2/loxP-UBC9-loxP-LEU2 bna7::KanMx/bna7::KanMx GPD-URA3-chr1/GPD-URA3-chr1
AB27-19	BNA2-OE bna7Δ	MATa/@ his3Δ1/his3Δ1 leu2Δ0/leu2Δ0 ura3Δ0/ura3Δ0 lys2Δ0/+ hoΔ::SCW11pr-Cre-EBD78-NATMX/hoΔ::SCW11pr-Cre-EBD78-NATMX loxP-CDC20-Intron-loxP-HPHMX/loxP-CDC20-Intron-loxP-HPHMX loxP-UBC9-loxP-LEU2/loxP-UBC9-loxP-LEU2 bna7::KanMx/bna7::KanMx GPD-BNA2-URA3-chr1/GPD-BNA2-URA3-chr1
UCC4925	Original control strain	MATa/MATα his3Δ1/his3Δ1 leu2Δ0/leu2Δ0 ura3Δ0/ura3Δ0 lys2Δ0/+ trp1Δ63/+ hoΔ::SCW11-cre-EBD78-NatMX/hoΔ::SCW11-cre-EBD78-NatMX loxP-CDC20-Intron-loxP-HphMX/loxP-CDC20-Intron-loxPHphMX loxP-UBC9-loxp-LEU2/loxP-UBC9-loxp-LEU2

Supplementary Table 2 (Primers)

Gene Knockout Primers using pRS400 as template (KO); Gene tagging primers using pKT-mCherry-WSB85 as template (tag); Restore gene using BY4743 as template (KI); pAG306GPD-xsome1 integration vector (OE)

ORF	Gene	Forward Primer	Reverse Primer	Forward Check Primer	Reverse Check Primer	Purpose
YDR127W	ARO1	ctagttgagaaatccctacgtaagataattgtatattacgctgtgcggtatttcacaccg	aagcattgtaaaatataaaaaaggatagatatattattgtagattgtactgagagtgcac	aactggtgagatcattggatgatgattgcc	ctcgaaacgtgagtcttttcc	KO
YJR078W	BNA2	catatataaagcgaaaataaaagaaagagtcaacgccgatctgtgcggtatttcacaccg	aaaaaaaaaaaaaaggggggcgttaataaaatgtataatgagattgtactgagagtgcac	aacacccgatactggtgacagggctatcta	ctcgaaacgtgagtcttttcc	KO
YFR047C	BNA6	gaacgcaatcctcagaaagaacacacaccaaaaaaaaaaactgtgcggtatttcacaccg	atgtaaataaaaaaaagtaaaaatcgaagcctaagcatttagattgtactgagagtgcac	atacctaatgtcaggtacataatgccagcg	ctcgaaacgtgagtcttttcc	KO
YJR025C	BNA1	taactgaaatagcatacatttaccaaaaaaaatcgaaaaaactgtgcggtatttcacaccg	taactgaaatagcatacatttaccaaaaaaaatcgaaaaaactgtgcggtatttcacaccg	aagctaccgtaattcatgcgtacagtaact	ctcgaaacgtgagtcttttcc	KO
YLR231C	BNA5	gcctaaatattctgtggaaaaaagaaacaaactggtaattctgtgcggtatttcacaccg	atatccaaaagaagatgaaggcgatgcggtcactctaggaagattgtactgagagtgcac	tctctcatatccagacgaagcaattgatgc	ctcgaaacgtgagtcttttcc	KO
YJL060W	BNA3	tactataattgcgtatagaatccattgttacttgctctcactgtgcggtatttcacaccg	tctatatattgagaaaaaggagtataaaaaaaaatttatcagattgtactgagagtgcac	atcgtctcctatgccaagataattactggc	ctcgaaacgtgagtcttttcc	KO
YML008C	ERG6	cgccgaaaccccctcccaaacttcccaagaagcaactcaaggtgacggtgctggttta	atatcgtgcgctttatttgaatcttattgatctagtgaattcgatgaattcgagctcg	tcccttggtattacccattaactggtgagt	cgcatgaactccttgatgatgg	tag
YDR007W	TRP1	gacgccagatggcagtagt	gtctccacacctccgcttac	tgcagcttcacagaaacctc	tctccacacctccgcttac	KI
YDR428C	BNA7	aaaaatcaaagcaaggaaaagctcagaggctgtcgtgaatctgtgcggtatttcacaccg	cacgcacacatatatataaatgcagaaataaaatcaactaagattgtactgagagtgcac	ttgagagaagagttattgttcccattcgca	ctcgaaacgtgagtcttttcc	
Integration plasmid	Chromosome 1			cgtggtttcttcgtggttt t	ataataccgcgccacatagc	OE
