# Supplementary material for: Independent regulation of age associated fat accumulation and longevity
Source: Nat Commun. 2020 Jun 3;11:2790. doi: 10.1038/s41467-020-16358-7 (PMC7270101; doi:10.1038/s41467-020-16358-7)
Supplement: Supplementary file 3 — Description of Additional Supplementary Information [file 41467_2020_16358_MOESM3_ESM.pdf]

Description of Additional Supplementary Files

File Name: Supplementary Data 1

Description: Aging cell lipid droplet suppressor screen. Sheet a, UCC4925 (WT) diploid cells were individually transformed in 96-well format with 280 high-copy 2 µm plasmids from a tiled genomic DNA library as described in Methods. Each plasmid contained a unique sequence-verified genomic DNA fragment that expressed at least one gene (ORF and Common name provided) previously determined to affect lipid droplet morphology or glycerolipid metabolism and additional genes that were not previously determined to affect these processes. To identify genes that suppressed age-induced accumulation of lipid droplets, all plasmid-expressing strains were individually grown in 2 mL of yeast extract peptone 2% glucose (YEPD) and kanamycin at 30°C using the Mother Enrichment Program (see Methods). Cells were stained with BODIPY 493/503 (ThermoFisher #D3922) or Nile Red (Sigma #N3013), and imaged by fluorescence microscopy. Plasmid containing strains in which 50% of median age 16 (i.e., middle age) cells exhibited young-cell-like levels of lipid droplets were scored as "few and/or dim lipid droplets" in the Excel table. Sheet b, To confirm potential suppressor genes (e.g., BNA2), high-copy 2 µm plasmids were isolated from cells that scored as "few and/or dim lipid droplets" in "Sheet a", verified by sequencing, retransformed into cells, and rescreened as described above. Sheet c, Candidate genes were transferred using LR clonase from pDONR221 Gateway entry plasmids from a previously described collection (HIP) into pAG306-GPD-ccdB-xsome1 as described in Methods. Plasmids were integrated into chromosome 1 after NotI digestion, and strains were then aged, and examined by fluorescence microscopy as described in Methods. The integrated suppressor (BNA2) was confirmed by the ability to maintain young-celllike levels of lipid droplets ("few and/or dim lipid droplets") in at least 50% of median age 16 cells.
