# Supplementary material for: Independent regulation of age associated fat accumulation and longevity
Source: Nat Commun. 2020 Jun 3;11:2790. doi: 10.1038/s41467-020-16358-7 (PMC7270101; doi:10.1038/s41467-020-16358-7)
Supplement: Supplementary file 5 — Reporting Summary [file 41467_2020_16358_MOESM5_ESM.pdf]

nature research

Corresponding author(s): Anthony O Beas

Last updated by author(s): Apr 6, 2020

# Reporting Summary

Nature Research wishes to improve the reproducibility of the work that we publish. This form provides structure for consistency and transparency in reporting. For further information on Nature Research policies, see Authors & Referees and the Editorial Policy Checklist .

## Statistics

For all statistical analyses, confirm that the following items are present in the figure legend, table legend, main text, or Methods section.

| n/a | Confirmed | |
|---|---|---|
| ☐ | ☒ | The exact sample size ($n$) for each experimental group/condition, given as a discrete number and unit of measurement |
| ☐ | ☒ | A statement on whether measurements were taken from distinct samples or whether the same sample was measured repeatedly |
| ☐ | ☒ | The statistical test(s) used AND whether they are one- or two-sided<br>*Only common tests should be described solely by name; describe more complex techniques in the Methods section.* |
| ☒ | ☐ | A description of all covariates tested |
| ☐ | ☒ | A description of any assumptions or corrections, such as tests of normality and adjustment for multiple comparisons |
| ☐ | ☒ | A full description of the statistical parameters including central tendency (e.g. means) or other basic estimates (e.g. regression coefficient) AND variation (e.g. standard deviation) or associated estimates of uncertainty (e.g. confidence intervals) |
| ☐ | ☒ | For null hypothesis testing, the test statistic (e.g. $F$, $t$, $r$) with confidence intervals, effect sizes, degrees of freedom and $P$ value noted<br>*Give P values as exact values whenever suitable.* |
| ☒ | ☐ | For Bayesian analysis, information on the choice of priors and Markov chain Monte Carlo settings |
| ☒ | ☐ | For hierarchical and complex designs, identification of the appropriate level for tests and full reporting of outcomes |
| ☒ | ☐ | Estimates of effect sizes (e.g. Cohen's $d$, Pearson's $r$), indicating how they were calculated |

*Our web collection on statistics for biologists contains articles on many of the points above.*

## Software and code

Policy information about availability of computer code

| | |
|---|---|
| Data collection | Fluorescent images of cells and lipid droplets were collected using a Leica microscope running Leica Application Suite X 3.3.3.16958 software; similarly, budscar counts for replicative lifespan analysis were collected on the same microscope. Flow cytometry data was collected using the Becton Dickinson (BD) FACSCanto II running BD FACSDiva 6.1.3 software. Global metabolomics data was collected using Thermo Xcalibur 4.0 (version 4.0.27.10) with Thermo Foundation 3.1 SP1 (version 3.1.83.0). Gas spectrometry mass spectrometry lipid data was collected using Agilent MSD Chemstation. Targeted metabolomics data was collected using Agilent MassHunter Data Acquisition and Processing. |
| Data analysis | Fluorescent images were analyzed using Adobe Photoshop CC 2019 v20.0.8. Replicative lifespans were analyzed and plotted using Microsoft Excel v16.34 and Graphpad Prism v8.3.1. Flow cytometry data were analyzed and plotted using FlowJo v10.6.1, Microsoft Excel v16.34 and Graphpad Prism v8.3.1. Global metabolomics data RAW files were converted to mzXML files using msconvert from the open source software package ProteoWizard, version 3.0.8789 (http://proteowizard.sourceforge.net/); peak identification and relative quantification were performed using the open source software program Maven, version 8.0.2 (https://github.com/eugenemel/maven); quantified measurements were analyzed and plotted using Microsoft Excel v16.34 and Graphpad Prism v8.3.1. Gas chromatography mass spectrometry lipids were analyzed and quantified using Agilent ChemStation Integrator, and measurements were analyzed and plotted using Microsoft Excel v16.34 and Graphpad Prism v8.3.1. Targeted metabolomics data were analyzed and quantified using Agilent MassHunter Data Acquisition and Processing, and then analyzed and plotted using Microsoft Excel v16.34 and Graphpad Prism v8.3.1. |

For manuscripts utilizing custom algorithms or software that are central to the research but not yet described in published literature, software must be made available to editors/reviewers. We strongly encourage code deposition in a community repository (e.g. GitHub). See the Nature Research guidelines for submitting code & software for further information.

## Data

Policy information about availability of data

All manuscripts must include a data availability statement. This statement should provide the following information, where applicable:

- Accession codes, unique identifiers, or web links for publicly available datasets
- A list of figures that have associated raw data
- A description of any restrictions on data availability

The source data for all four figures and four supplemental figures are provided in a single “source data” file accompanying this manuscript. The metabolomics datasets are included in this source data file.

# Field-specific reporting

Please select the one below that is the best fit for your research. If you are not sure, read the appropriate sections before making your selection.

☒ Life sciences ☐ Behavioural & social sciences ☐ Ecological, evolutionary & environmental sciences

For a reference copy of the document with all sections, see nature.com/documents/nr-reporting-summary-flat.pdf

# Life sciences study design

All studies must disclose on these points even when the disclosure is negative.

**Sample size**

Because our fluorescence imaging revealed visually that lipid droplets accumulate during aging, we designed a high throughput, flow cytometry-based method to measure the levels of cellular lipid droplets in hundreds of thousands of cells (total) over multiple strains and at least four independent experiments per figure panel presented. Based on our fluorescence and flow cytometry measurements, we carried out gas chromatography mass spectrometry (five independent experiments) to biochemically analyze neutral lipids of lipid droplets in young and aged cells, and this independently complimented our fluorescence and flow cytometry findings.

For replicative lifespan studies, we have previously determined that from ~20 cells we can use the gold standard microdissection method to measure and determine statistically significant differences in replicative lifespan between samples. In this study, we used microdissection to reveal a statistically significant difference in replicative lifespan between control and BNA2-OE cells, and developed a novel, more high throughput method (based our previously characterized Mother Enrichment Program (itself shown to be able to distinguish between the replicative lifespans of different yeast strains) and budscar counting) to perform numerous, independent experiments, with at least one experiment performed by a different team member, analyzing >2000 control and BNA2-OE cells total per strain. Our novel method yielded a very similar significant difference in replicative lifespan between control and BNA2-OE strains as with microdissection. Based on these findings, we used our novel method to assess differences in lifespan in other strains by analyzing at least 325 cells total cells from at least three independent experiments were performed, and one of three experiments was performed by a different team member, to determine if there were statistically significant differences in replicative lifespan between yeast strains.

For our global metabolomics data, we analyzed at least three independent experiments per strain. Based on our findings with global metabolomics, we identified trends of interest, and based on these trends, we used a targeted based approach (targeted metabolomics) to analyze specific metabolites of interest in four independent experiments per strain. Combined, we performed seven metabolomics experiments for control and BNA2-OE strains using complimentary methods, and these methods yielded highly complementary findings.

**Data exclusions**

No data were excluded from our analyses.

**Replication**

In order to achieve statistical robustness, we performed a minimum of 3 biological replicates. All attempts at replication were successful. Importantly, different team members performed independent experiments for some of the results presented in the figure panels, to increase the confidence that our findings are robust and significant and to minimize the potential for bias.

**Randomization**

Randomization was not relevant to our studies. All data presented in each figure panel represents strains (mutants) that were aged, grouped and analyzed in the same set of independent experiments and compared within that same group in order to determine if there were or were not relevant differences in lifespan, lipid droplet levels, metabolites levels, etc. among those grouped strains.

**Blinding**

In general, blinding was not applied because the differences in the phenotypes we analyzed were robust/significant, and would only marginally be affected by blinding. Importantly, different investigators performed and scored different biological replicates, which provided an independent means of assessing scoring accuracy and minimizing the potential for bias.

# Reporting for specific materials, systems and methods

We require information from authors about some types of materials, experimental systems and methods used in many studies. Here, indicate whether each material, system or method listed is relevant to your study. If you are not sure if a list item applies to your research, read the appropriate section before selecting a response.

## Materials & experimental systems

| n/a | Involved in the study |
|---|---|
| ☒ | ☐ Antibodies |
| ☒ | ☐ Eukaryotic cell lines |
| ☒ | ☐ Palaeontology |
| ☒ | ☐ Animals and other organisms |
| ☒ | ☐ Human research participants |
| ☒ | ☐ Clinical data |

## Methods

| n/a | Involved in the study |
|---|---|
| ☒ | ☐ ChIP-seq |
| ☐ | ☒ Flow cytometry |
| ☒ | ☐ MRI-based neuroimaging |

# Flow Cytometry

## Plots

Confirm that:

☒ The axis labels state the marker and fluorochrome used (e.g. CD4-FITC).

☒ The axis scales are clearly visible. Include numbers along axes only for bottom left plot of group (a 'group' is an analysis of identical markers).

☒ All plots are contour plots with outliers or pseudocolor plots.

☒ A numerical value for number of cells or percentage (with statistics) is provided.

## Methodology

Sample preparation: For each experiment, a cohort of mother yeast cells were aged in cultures, and at each aging time point represented in each figure panel, a sample of the original cohort was obtained, fixed, and processed for flow cytometry according to the methods.

Instrument: Becton Dickinson FACS Canto II running FACSDiva 6.1.3 software

Software: Flow cytometry data were analyzed using FlowJo v10.6.1, Microsoft Excel v16.34 and Graphpad Prism v8.3.1

Cell population abundance: In order to determine that we were quantifying lipid droplets in live, aged, mother yeast cells, we labeled an original cohort of mother yeast cells with magnetic beads to allow for enrichment of these original labeled mother cells from cultures at various time points using magnets. We then labeled the magnetically enriched cells at each aging time point with Wheat Germ Agglutinin Alexa Fluo 647 Conjugate, a highly specific budscar label, which we used to gate aged mother cells that became brighter and brighter on the cell surface as they gained budscars during replicatively aging in culture, we used SYTOX Blue Dead Cell Stain to gate for live, stain negative aged cells, and then stained with BODIPY 493/503, a highly specific neutral lipid stain of lipid droplets to measure lipid droplet levels. We independently visually checked the quality of samples analyzed by flow cytometry using fluorescence microscopy.

Gating strategy: After enriching for aged cells using magnetic beads (>85% purity), we first used forward and side scatter to gate by size (mother cells are known to be larger than newborn daughter cells), we used a label for budscars to gate for aged versus newborn daughters (which have no budscars -- hence no budscar labeling), we used a live dead stain that permeates only dead cells to gate out live (stain negative) cells, and finally we measured a specific, well characterized lipid droplet stain in order to quantify lipid droplets in original, aged, live mother yeast cells at each time point. An example of our gating strategy is provided in supplemental Figure 1.

☒ Tick this box to confirm that a figure exemplifying the gating strategy is provided in the Supplementary Information.
